# Identification of Immune Cells and Key Genes associated with Alzheimer's Disease

**DOI:** 10.7150/ijms.66422

**Published:** 2022-01-01

**Authors:** Chenming Liu, Xi Zhang, Huazhen Chai, Sutong Xu, Qiulu Liu, Yuping Luo, Siguang Li

**Affiliations:** 1 Key Laboratory of Spine and Spinal Cord Injury Repair and Regeneration of Ministry of Education, Orthopedic Department of Tongji Hospital, Tongji University School of Medicine, Shanghai, China.; 2 Stem Cell Translational Research Center, Tongji Hospital, Tongji University School of Medicine, Shanghai, China.

**Keywords:** Alzheimer's Disease, immune infiltration, bioinformatics, key genes

## Abstract

Alzheimer's disease (AD) is an age-related neurodegenerative disorder characterized by cognitive impairment and memory loss, for which there is no effective cure to date. In the past several years, numerous studies have shown that increased inflammation in AD is a major cause of cognitive impairment. This study aimed to reveal 22 kinds of peripheral immune cell types and key genes associated with AD. The prefrontal cortex transcriptomic data from Gene Expression Omnibus (GEO) database were collected, and CIBERSORT was used to assess the composition of 22 kinds of immune cells in all samples. Weighted gene co-expression network analysis (WGCNA) was used to construct gene co-expression networks and identified candidate module genes associated with AD. The least absolute shrinkage and selection operator (LASSO) and random forest (RF) models were constructed to analyze candidate module genes, which were selected from the result of WGCNA. The results showed that the immune infiltration in the prefrontal cortex of AD patients was different from healthy samples. Of all 22 kinds of immune cells, M1 macrophages were the most relevant cell type to AD. We revealed 10 key genes associated with AD and M1 macrophages by LASSO and RF analysis, including *ARMCX5*, *EDN3*, *GPR174*, *MRPL23*, *RAET1E*, *ROD1*, *TRAF1*, *WNT7B*, *OR4K2* and* ZNF543*. We verified these 10 genes by logistic regression and k-fold cross-validation. We also validated the key genes in an independent dataset, and found *GPR174*, *TRAF1*, *ROD1*, *RAET1E*, *OR4K2*, *MRPL23*, *ARMCX5* and *EDN3* were significantly different between the AD and healthy controls. Moreover, in the 5XFAD transgenic mice, the differential expression trends of *Wnt7b*, *Gpr174*, *Ptbp3*, *Mrpl23*, *Armcx5* and *Raet1e* are consistent with them in independent dataset. Our results provided potential therapeutic targets for AD patients.

## Introduction

Alzheimer's disease (AD) is one of the most common neurodegenerative diseases with a high prevalence 1, which is currently difficult to cure and imposes a heavy burden on both patients and families 2. The main clinical manifestations of AD patients are memory loss and cognitive dysfunction, and its pathology is mainly characterized by Aβ deposition 3, tau protein hyperphosphorylation 4, neurofibrillary tangles 5 and an increase in inflammation 6.

A growing evidence suggests that neuroinflammation is a major player in AD pathology. Microglia and astrocytes are actively involved in the immune response of the Central Nervous System (CNS) 7, 8. During the development of AD, microglia enter an activated state 9, which may affect neuronal apoptosis and the maintenance of synaptic plasticity 10. Activated microglia internalize pathogenic substances in the brain through cytosolic drinking, phagocytosis or receptor-mediated endocytosis 11-13. However, microglia in the aging brain are subject to dysfunction or persistent activation 14-16, which usually leads to an exacerbation of AD pathology. Transcriptome studies have revealed that activated astrocytes also exist in AD 17. It has been proposed that inflammatory injury induces the A1 astrocyte through the NF-κB pathway. What's more, astrocytes of the A1 phenotype can express inflammatory mediators 18. The homeostatic function of astrocytes is affected in in neurodegenerative disorders which induces the death of neurons and oligodendrocytes 19. Moreover, activated microglia and astrocytes could interact and promote neurodegeneration 20.

Several recent studies have highlighted the close association of AD and peripheral inflammation 21. For example, the presence of pro-inflammatory cytokines may increase the probability of AD in obese people 22, and obesity has been listed as one of the risk factors for AD 23, 24. Strong evidence showed that type II diabetes increases the risk of AD 25. Moreover, peripheral immune cells, including macrophages, natural killer (NK) cells and neutrophils, are actively involved in the pathological response to AD 26. Monocyte chemokine receptors, such as CXCL1, are more highly expressed in AD patients 27. And neutrophils can enter the CNS of AD mice and surround Aβ plaques via neutrophil extracellular traps 28, which may lead to increased neuroinflammation 29. In addition, T regulatory cells (TRegs) are at high peripheral level in AD patients 30, and the increase of TRegs could be beneficial to AD patients, which affect microglia responses 31. In conclusion, immune cells are involved in the occurrence of AD. The molecular mechanisms between immune cells and the occurrence of AD needs to be further investigated.

In this study, we explored the relationship between immune cells and AD and identified AD-associated key genes by a range of bioinformatics methods. We revealed the alteration of immune infiltration of AD pathology, explored AD-associated immune cells, and identified AD-related key genes which affected the occurrence and development of AD. Our results refined the current knowledge on immune infiltration of AD pathology and provided a valuable resource for future studies on immune-related signaling pathways in AD.

## Methods and Materials

### Data preprocessing and immune cell evaluation

**Figure 1:**
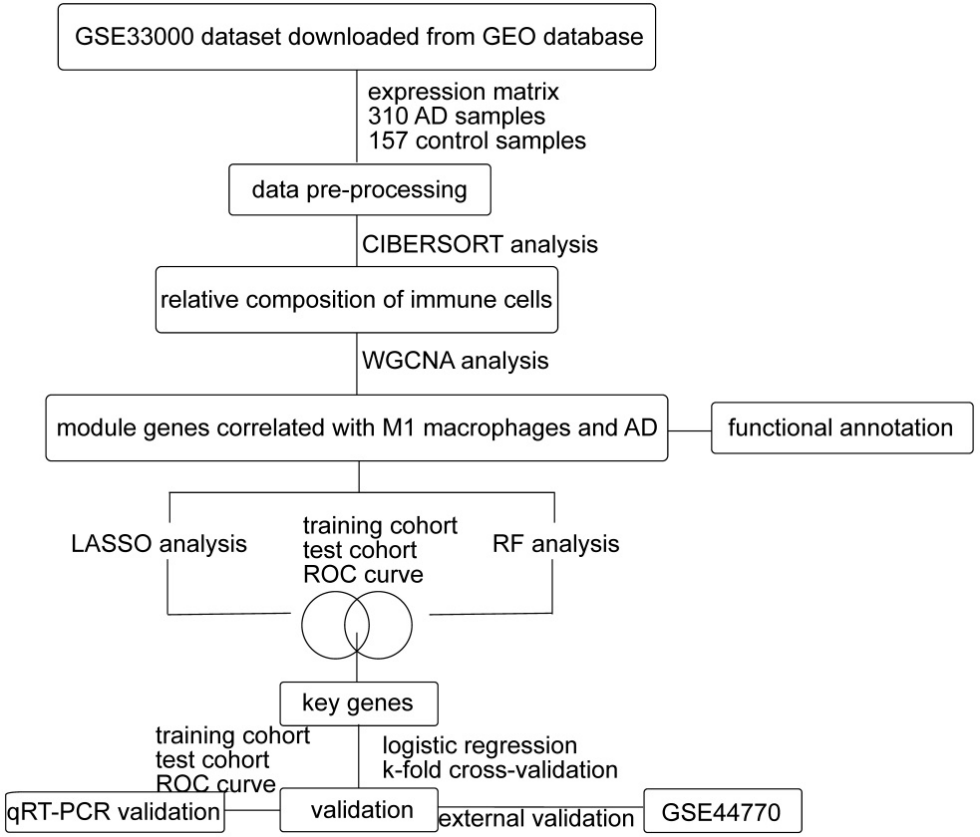
** Flow chart of this study.** GEO: Gene Expression Omnibus, AD: Alzheimer's Disease, WGCNA: weighted gene co-expression network analysis, LASSO: least absolute shrinkage and selection operator, RF: random forest, ROC: receiver operating characteristic.

As shown in Figure 1, we first downloaded the GSE33000 dataset from Gene Expression Omnibus (GEO) database. The dataset including 310 samples from the prefrontal cortex of AD patients and 157 samples from healthy controls. We used R software to process the raw data of the GSE33000 dataset. Then the raw matrix was normalized by the limma package (version 3.46.0) 32. CIBERSORT was performed to identify the composition of 22 immune cells from gene expression profiles 33. If the immune cell type is not detected in more than half of all samples, this cell type will be excluded. The retained cell types could be detected in most samples. We determined the differential immune cells between two groups (*p*-value < 0.05) by wilcox.test. The level of immune cell infiltration was demonstrated using ggplot2 (version 3.3.3) and pheatmap (version 1.0.12) packages. As for external verification, we downloaded the GSE44770 dataset, which includes 129 AD samples and 101 healthy controls of the prefrontal cortex. The raw data of the GSE44770 dataset were also processed in the same way of GSE33000 dataset.

### WGCNA

Firstly, the raw expression matrix of the dataset was normalized by the limma package, and genes in the top 25% variance of all samples were screened, then gene co-expression networks were constructed using WGCNA package (version 1.70-3) 34. We first determined an appropriate soft threshold power to achieve a scale-free topology. By calculating the similarity between genes, the coefficient of similarity between genes was obtained, and genes were clustered into different modules and labeled with different colors. We set the minimum number of genes per module to 30. Trait information was based on the outcome of immune infiltration and disease. Correlations between modules and the trait information were calculated through Pearson correlation method. The module with the highest correlation with traits was selected for subsequent analysis. Then we performed further screening based on gene significance (GS) and module importance (MM).

### Functional enrichment analysis

The Kyoto Encyclopedia of Genes and Genomes (KEGG) and Reactome pathway analysis of key module genes were performed by clusterProfiler (version 3.18.1) 35 and ReactomePA 36 (version 1.34.0) packages. The significance criterion was *p*-value<0.05.

### Construction of models and identification of key genes

To filter out the key genes, we analyzed the selected candidate module genes using the least absolute shrinkage and selection operator (LASSO) and random forest (RF), which were used to calculate the importance of key genes in the dataset. The “randomForest” (version 4.6-14) and “glmnet” (version 4.1-1) packages were adopted for LASSO and RF analysis 37. The data were randomly divided into training cohort and test cohort, and the intersection of the two gene lists from the training cohort by LASSO and RF analysis was regarded as the key genes. We used VennDiagram (version 1.6.20) 38 to visualize the intersection of gene lists. The sensitivity and specificity of the model were validated by receiver operating characteristic (ROC) curve using “ROCR” package (version 1.0-11) 39.

### Validation of key genes in GEO datasets

In order to validate the relationship between AD and key genes, we constructed logistic regression model based on the common genes of the two gene lists in the training cohort. The internal data GSE33000 were randomly grouped according to the test cohort and training cohort 3/7. We generated logistic regression model in the training cohort, and the test cohort was used for verification. The ROC curve of the pROC (version 1.17.0.1) package was used to evaluate the effectiveness of the model 40. Confusion matrix evaluated the accuracy of the model. In addition, we also use k-fold cross-validation to verify our results. The samples of the GSE33000 dataset were randomly divided into 10 equal parts, each time 1 of the 10 parts was used as the test cohort, and all the others were used as the training cohort. The maximum test cohort accuracy and training cohort accuracy were found for ten cross-validations, the fold corresponding to the maximum accuracy was used as the test cohort and the rest as the training cohort. The ROC and confusion matrix were also used to evaluate the effectiveness of the model in the test and training cohort. We also verified the relative expression levels of key genes in the GSE44770 dataset. The ggboxplot was used to display the relative expression levels of key genes in the AD and control groups. We used T-test for statistical analysis between two groups. The significance criterion was *p*-value<0.05.

### Animals

5.5-month-old heterozygous 5XFAD mice (on a C57BL/6N background) overexpress mutant human amyloid beta (A4) precursor protein 695 (APP) with the Swedish (K670N, M671L), Florida (I716V), and London (V717I) Familial Alzheimer's Disease (FAD) mutations along with human presenilin 1 (PS1) harboring two FAD mutations, M146L and L286V. Both transgenes are regulated by the mouse Thy1 promoter to drive overexpression in the brain 41. The mice are kept in Tongji University Animal Center with constant temperature and humidity, light and dark cycle for 12h. The control group and the AD group consist of two male mice and two female mice, respectively.

### RNA extraction and quantitative real time-PCR

We extract RNA from the prefrontal cortex of all mice by RNAiso Plus (9109, TaKaRa, Dalian, China) following the manufacturer's instructions. Quantitative real-time PCR was carried out using the AceQ Universal SYBR qPCR Master Mix (Q411, Vazyme, Biotech, Nanjing, China). Relative expression levels of genes were calculated by ΔΔCt method and normalized to β-Actin and compared with control samples. All sequences for RNA primers are shown in Table 1.

### Statistical analysis

All data are presented as mean ± standard deviation (SD). Each experiment was replicated at least three times. Data visualization and analysis were performed with GraphPad Prism 8 (GraphPad Software Inc., La Jolla, CA, USA). Statistical analysis was performed using student's t-test.

## Results

### Immune infiltration was altered in the prefrontal cortex of AD patients

**Figure 2:**
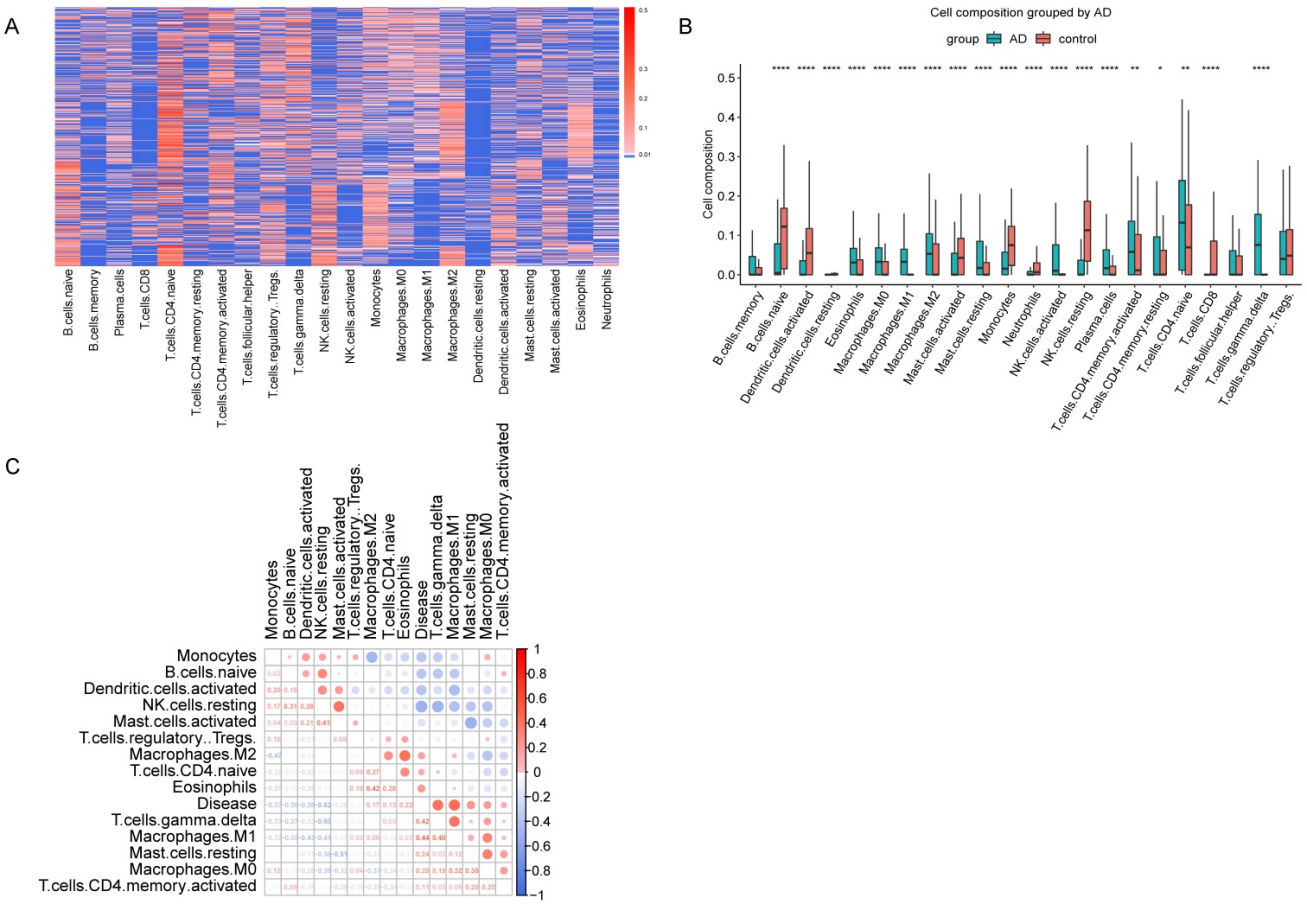
** Immune Infiltration Analysis. (A)** Heatmap showed the composition of 22 kinds of immune cells in each sample. The relative composition was higher from blue to red. Each row in the heatmap was a sample. **(B)** Boxplot of the composition of immune cells. **(C)** Correlation matrix of 14 immune cells and disease. Red represents positive correlation, and blue represents negative correlation.

GSE33000 dataset include 310 AD samples and 157 healthy samples from the prefrontal cortex. CIBERSORT was performed to obtain the relative composition of 22 kinds of immune cells (Figure 2A). The composition of the 22 kinds of immune cells was shown in Figure 2B, and the immune infiltration was different in AD and healthy samples. As described in materials and methods, we found 8 cell types, memory B cells, resting dendritic cells, neutrophils, activated NK cells, plasma cells, resting memory CD4 T cells, CD8 T cells and follicular helper T cells were not detected in more than half of all samples, so we excluded these cell types, and the remained 14 cell types were used for subsequent analyses. Pearson correlation coefficients were used to calculate the correlation of the 14 immune cell types with AD. As shown in Figure 2C, M2 macrophages, CD4 naive T cells, regulatory T cells, eosinophils, gamma delta T cells, M1 macrophages, resting mast cells, M0 macrophages and activated CD4 memory T cells are positively correlated with AD.

### M1 macrophages were highly associated with AD

**Figure 3:**
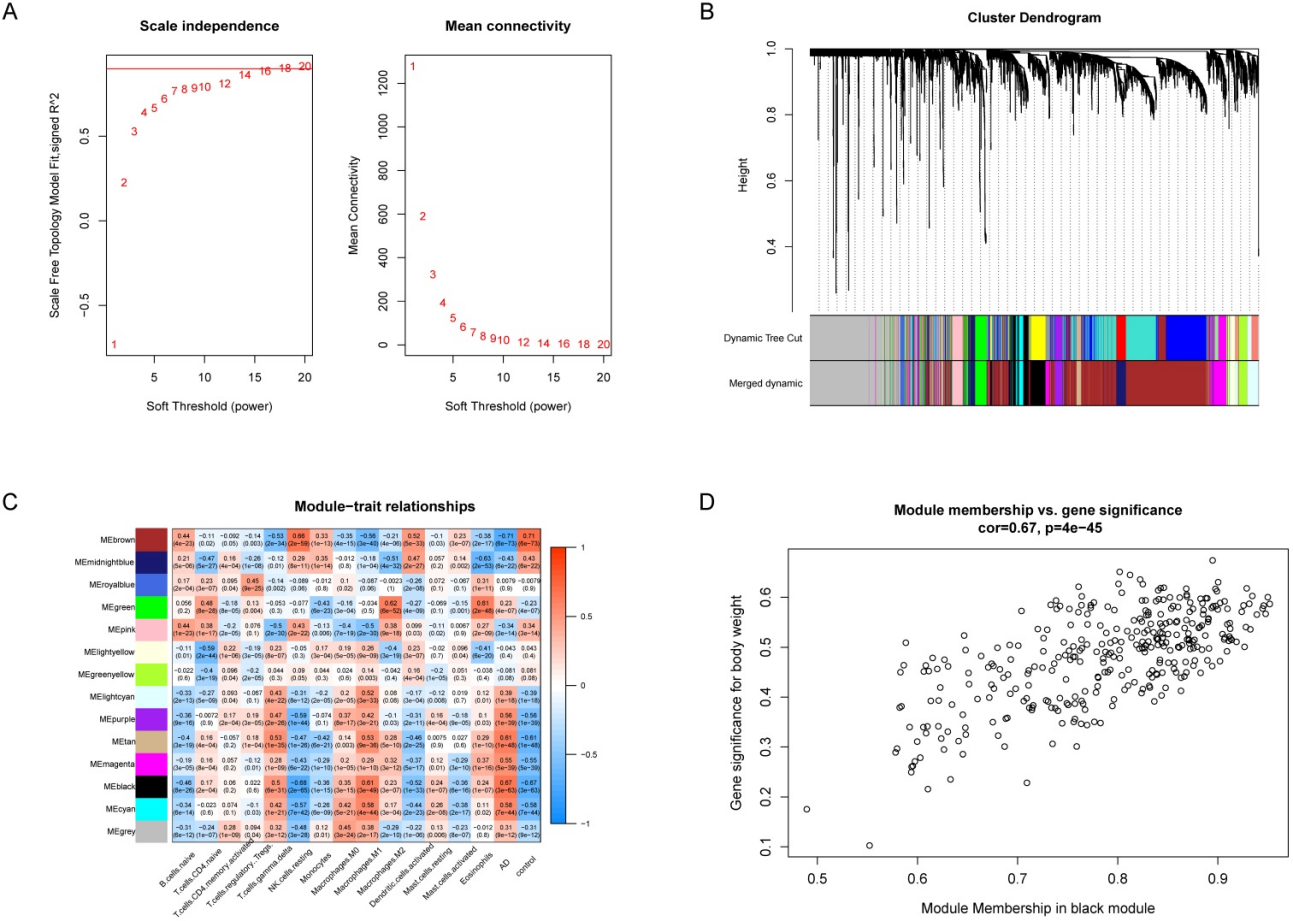
** WGCNA revealed gene co-expression networks and module-trait relationships. (A)** Selecting the best soft threshold power β. **(B)** Dendrogram of top 25% variance genes. **(C)** The heatmap of module-trait relationships. The black module had the strongest correlation with M1 macrophages.** (D)** The scatterplot of gene significance (GS) against module membership (MM) in the black module.

WGCNA was utilized to analyze the co-expression networks associated with immune cells in AD. We normalized the data of the GSE33000 dataset and subsequently screened the genes with the top 25% variance, and 4142 genes which meet the requirement were obtained. Then we chose soft threshold power β = 14 to build a scale-free co-expression network, the scale-free R^2^ > 0.85 (Figure 3A). Based on the similarity between genes, the 4142 genes were clustered into 14 different color modules (Figure 3B, 3C). Correlation analysis was performed between modules, the 14 retained immune cell types and disease status. The module exhibiting highest positive correlation with AD was black module, which contain 336 co-expression genes, and the immune cell exhibiting highest positive correlation with black module was M1 macrophages (Figure 3C), suggesting M1 macrophages were highly associated with AD.

### Genes in black module were mainly affect nucleotide excision repair, ion transport and Hedgehog signaling pathway

**Figure 4:**
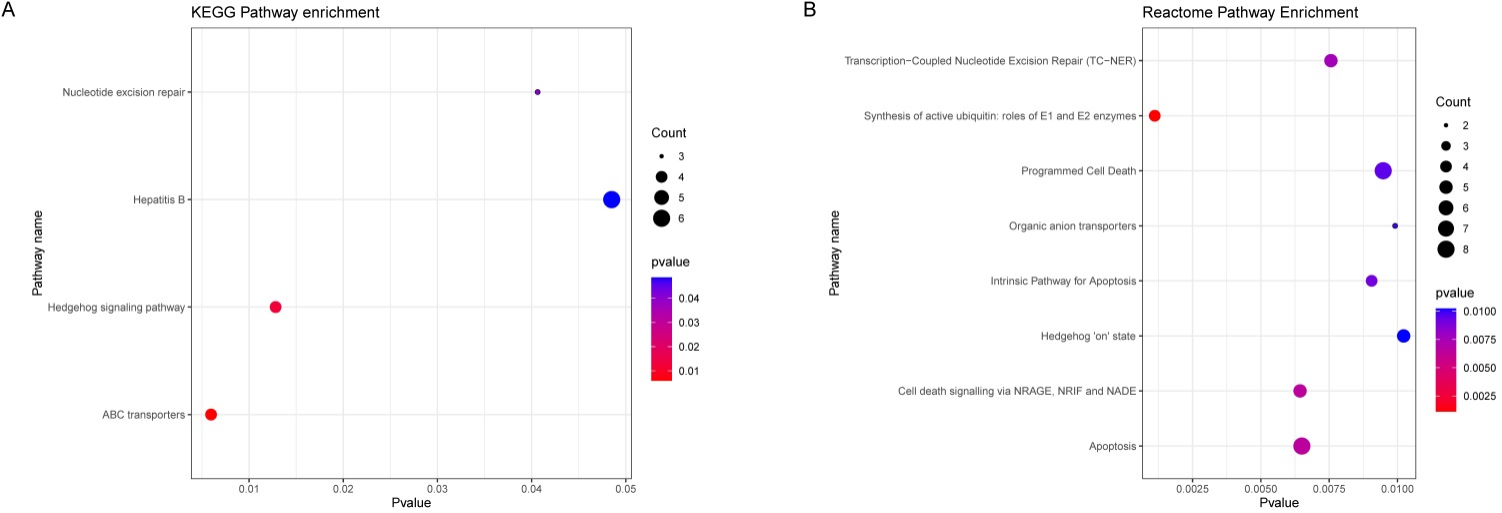
** Functional Enrichment Analysis. (A)** KEGG enrichment analysis of black module genes. **(B)** Reactome enrichment analysis of black module genes.

To further explore the potential biological pathways and processes about the black module genes, we performed KEGG and Reactome functional annotation analyses based on 336 black module genes (Figure 4A, 4B). The results of KEGG pathway analysis showed that black module genes were significantly enriched in nucleotide excision repair, Hepatitis B, Hedgehog signaling pathway and ABC transporters. The results of Reactome pathway analysis suggested that black module genes were mainly enriched in Transcription-Coupled Nucleotide Excision Repair (TC-NER), Synthesis of active ubiquitin: roles of E1 and E2 enzymes, Programmed Cell Death, Organic anion transporters, Intrinsic Pathway for Apoptosis, Hedgehog 'on' state, Cell death signaling via NRAGE, NRIF and NADE, Apoptosis. Based on the p-value and frequency of each term, these results suggested that the black module genes mainly affect nucleotide excision repair, ion transport and Hedgehog signaling pathway.

### Identification and validation of 10 genes associated with M1 macrophages and AD in GEO datasets

**Figure 5:**
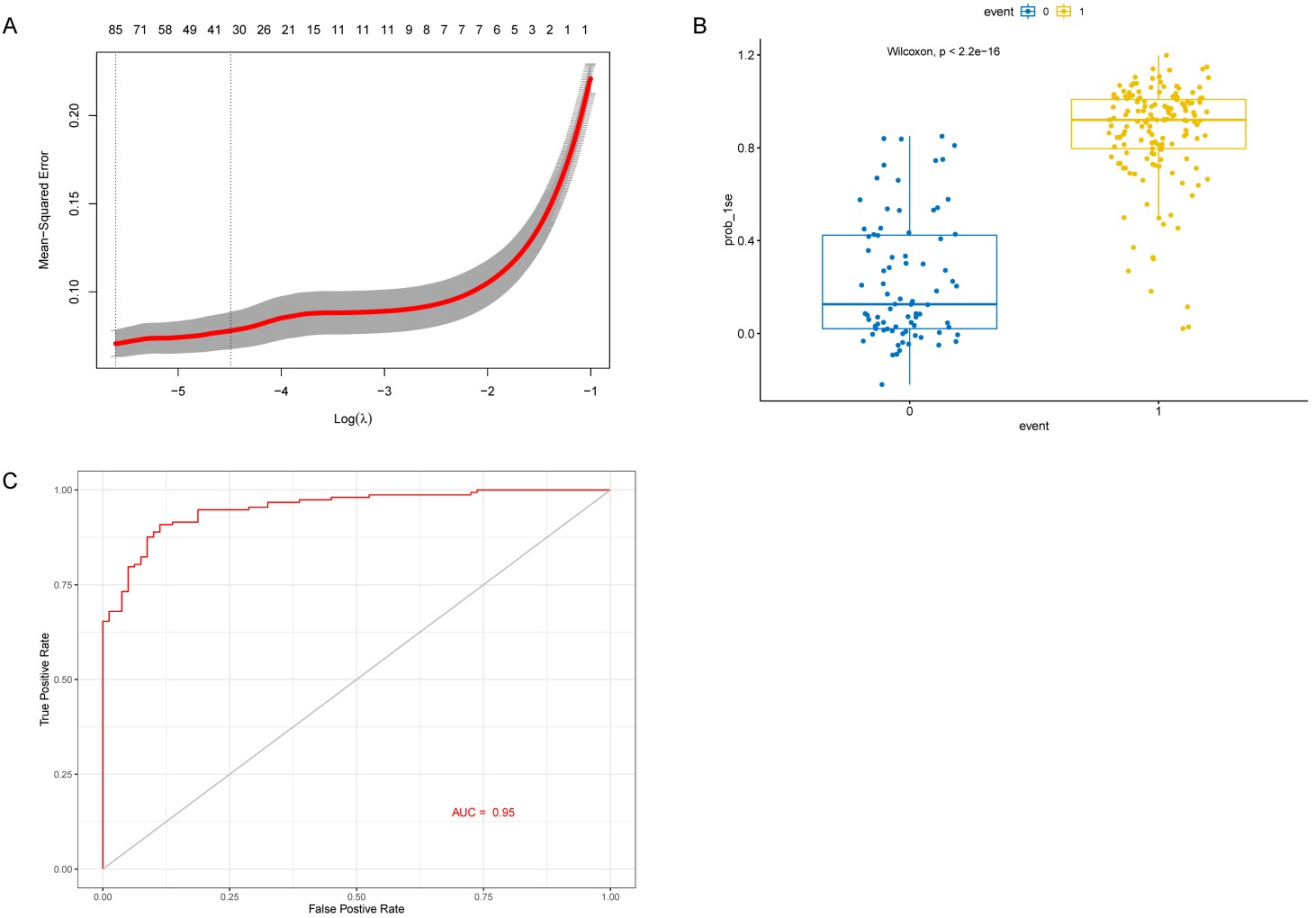
** Identification and verification 31 genes in the LASSO model. (A)** The partial likelihood deviance for the lasso regression. λ is the tuning parameter. **(B)** The LASSO model between AD samples and control samples in the test cohort. **(C)** ROC curve verified the effectiveness of LASSO model in the test cohort.

**Figure 6:**
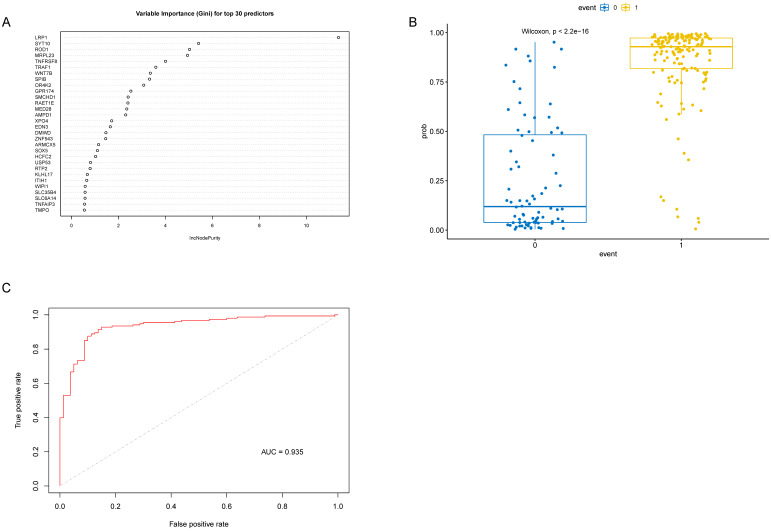
** Identification and verification 30 genes in the RF model. (A)** Top 30 genes based on variable importance in RF analysis. **(B)** The RF model between AD samples and control samples in the test cohort. **(C)** ROC curve of RF model in the test cohort.

**Figure 7:**
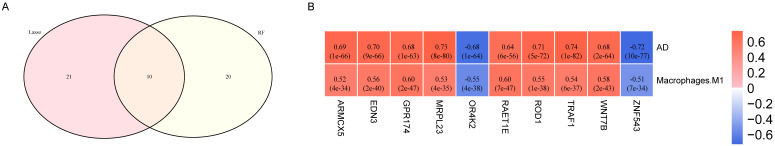
** Correlation analysis of key genes with M1 macrophage and AD. (A)** Venn diagram of the 10 communal key genes between Lasso and RF analysis. **(B)** The correlations between the common genes respectively identified by LASSO and RF analysis and M1 macrophage and AD. Blue represents negative correlation, and red represents positive correlation.

As the WGCNA results demonstrated, black module was the key module associated with M1 macrophages and AD. Based on the cut-off criteria (|MM| > 0.8 and |GS| > 0.2), 242 genes with high connectivity in the black module were selected for the LASSO and RF analysis (Figure 3D). According to the relationship between mean square error of cross-validated and model size, we generated the LASSO regression model based on the 1-se criteria (Figure 5A). The 1-se gives a model with excellent performance and a minimum number of independent variables, at which point 31 non-zero variables are retained. Table 2 showed the estimated coefficients between the LASSO regressions of genes screened by the black module and AD. As a result, 31 key genes were identified by LASSO analysis. What's more, the LASSO model based on expression levels of these genes was also different in AD and control in the test cohort (Figure 5B). Then we performed ROC curve in the test cohort to evaluate the effectiveness of the LASSO regression model. The area under the curve (AUC) was 0.95 (Figure 5C). The top 30 genes based on the parameter of increase in node purity in RF analysis were used for subsequence analysis (Figure 6A, Table 3). The RF model based on expression levels of the candidate genes was also different in AD and control samples in the test cohort (Figure 6B). The AUC of the RF model in the test cohort was 0.935 (Figure 6C). These all proved that our models were reliable for identifying genes which affect the occurrence of AD. Therefore, we defined the common genes of the two gene lists identified by random forest and LASSO regression respectively, as the key genes associated with M1 macrophages and AD (Figure 7A). Then correlation analysis showed 10 genes were key genes associated with M1 macrophages and AD, of which *ARMCX5*, *EDN3*, *GPR174*, *MRPL23*, *RAET1E*, *ROD1*, *TRAF1* and *WNT7B* were positively associated with M1 macrophages and AD, while* OR4K2* and* ZNF543* were negatively associated with M1 macrophages and AD (Figure 7B).

**Figure 8:**
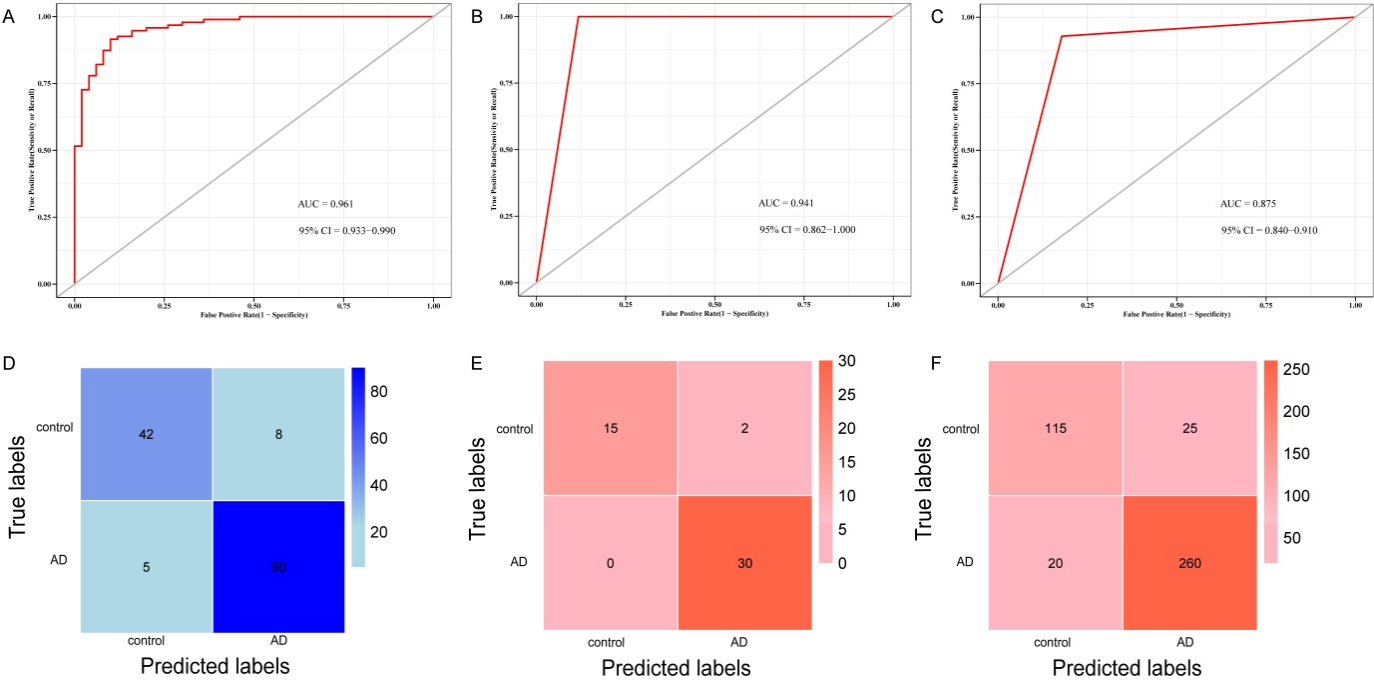
** ROC curve and confusion matrix of logistic model and k-fold cross-validation based on key genes. (A)** ROC curve for distinguishing AD from control in the test cohort of logistic model. **(B)** ROC curve for distinguishing AD from control in the test cohort of the k-fold cross-validation. **(C)** ROC curve for differentiating AD from control in training cohort of the k-fold cross-validation. **(D)** Confusion matrix of the logistic regression model in test cohort. **(E)** Confusion matrix of the k-fold cross-validation in test cohort. **(F)** Confusion matrix of the k-fold cross-validation in training cohort.

**Figure 9:**
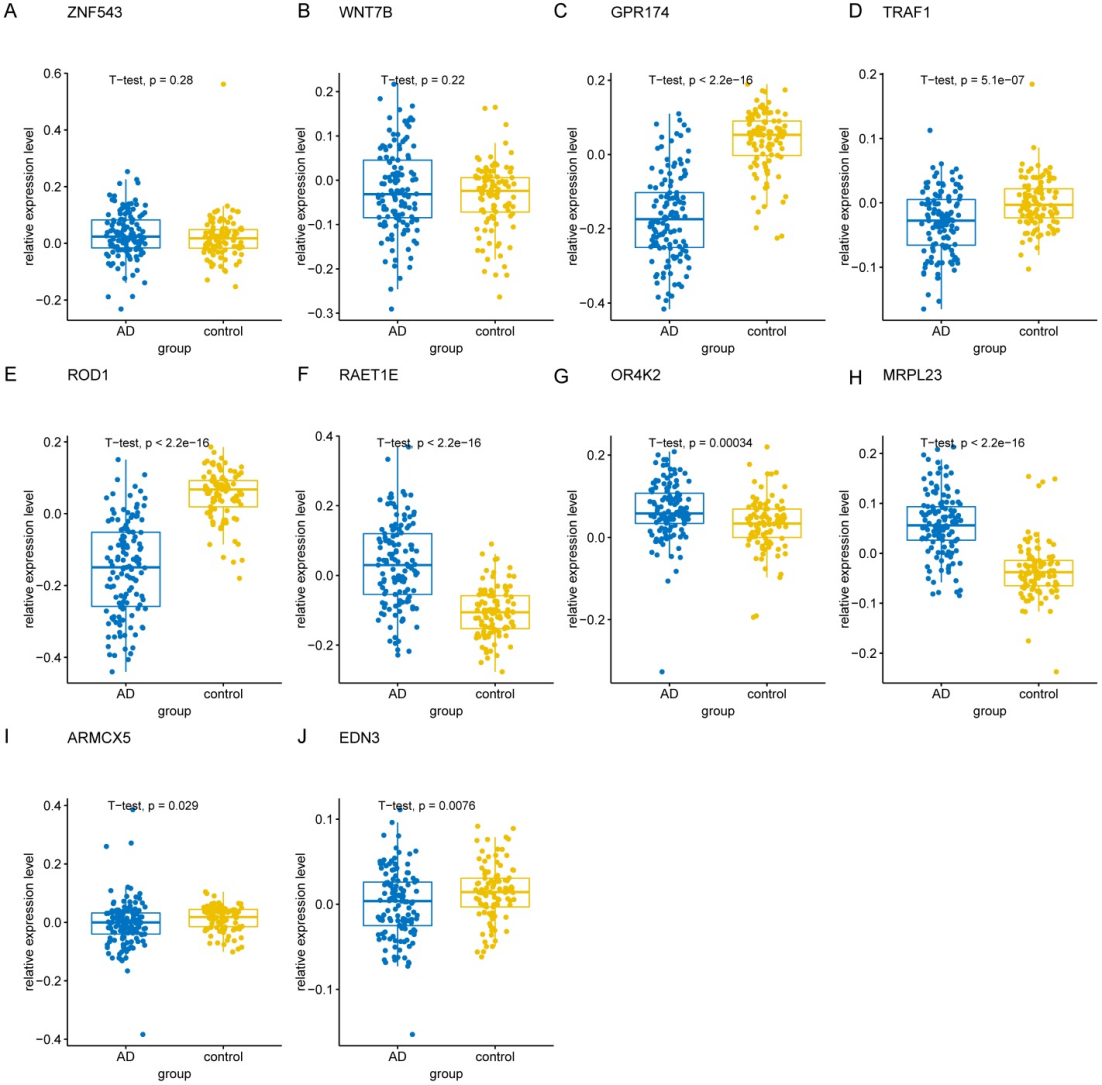
** The relative expression levels of 10 key genes in GSE44770 dataset. (A-J)** The boxplot showed the relative expression levels of 10 key genes in AD and healthy controls.

To validate the 10 genes associated with the occurrence of AD, we constructed logistic regression model based on the expression matrix of 10 key genes associated with AD in the training cohort, and validated them in the test cohort by ROC curve (AUC = 0.961, 95% CI=0.933-0-990) (Figure 8A). In addition, we also validated the genes using 10-fold cross-validation, we evaluated the model in the test and training cohort by ROC curve. For the 10-fold cross-validation, both the test (AUC = 0.941, 95% CI = 0.862-1) and training cohort (AUC = 0.875, 95% CI = 0.840-0.910) showed relatively good performance (Figure 8B-C). We also evaluated the models by confusion matrix in the test and training cohort (Figure 8D-F), the accuracy and recall of the models based on the confusion matrix of the test cohort and training cohort were shown in Table 4. All of these results showed that these genes were the key genes associated with the occurrence of AD. GSE44770 dataset was used as an independent dataset to verify the relative expression of 10 key genes in AD and healthy controls. We found that *GPR174, TRAF1, ROD1, RAET1E, OR4K2, MRPL23, ARMCX5* and *EDN3* were differentially expressed between the AD and healthy controls (*p*<0.05) (Figure 9A-J).

### Seven key homologous genes associated with human AD are differentially expressed between 5XFAD models and wild type mice

**Figure 10:**
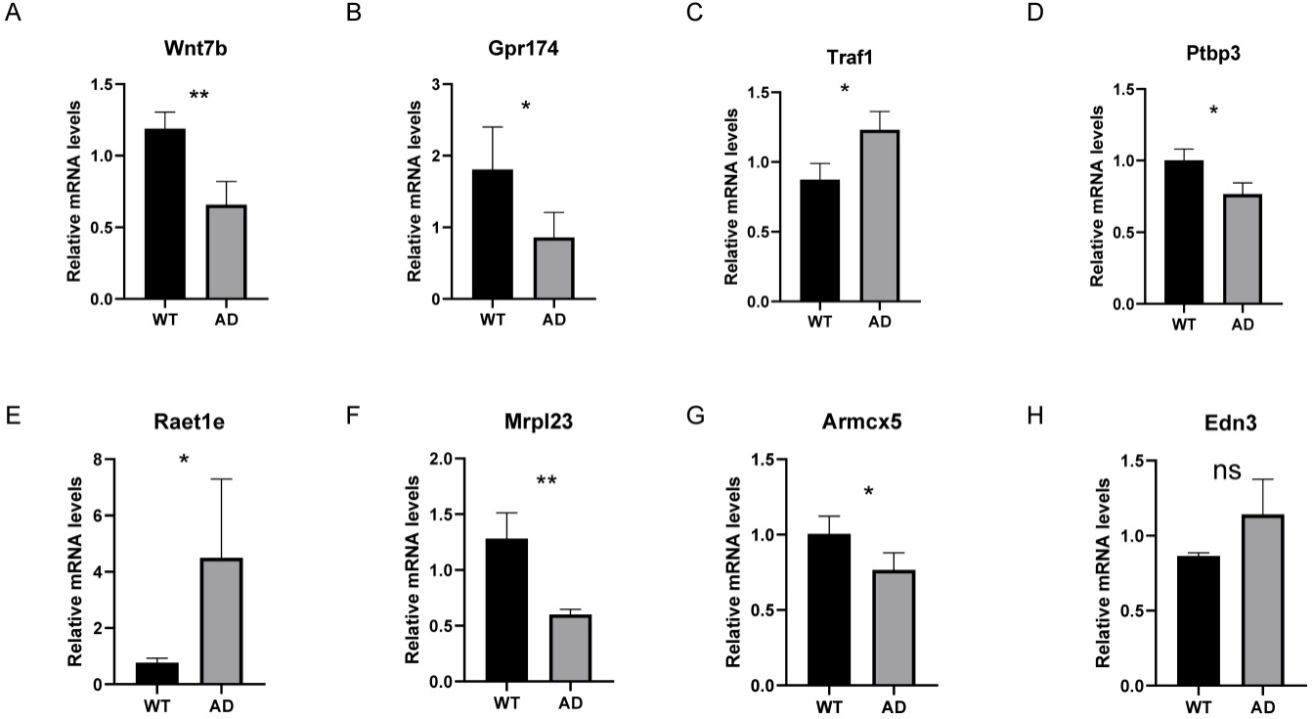
** The relative mRNA levels of 8 genes in AD mouse models. (A-H)** The relative mRNA levels of *Wnt7b*, *Gpr174*, *Traf1*, *Ptbp3*, *Raet1e*, *Mrpl23*, *Armcx5* and *Edn3* in control and 5XFAD samples compared with the control. ns P>0.05, *p < 0.05, **p < 0.01 and ***p < 0.001.

In addition to validating our results in an independent dataset, we also validated the common gene list of LASSO and RF analysis in the 5XFAD model. Since *ZNF543* and *OR4K2* have no homologs in mice, we examined the relative mRNA levels of the remaining 8 genes in mice. As illustrated in Figure 10, compared to the control group, the relative mRNA levels of *Traf1* and *Raet1e* were significantly increased compared to the control group (*P* < 0.05) and the AD group showed significantly decreased mRNA levels of *Wnt7b*, *Gpr174*, *Ptbp3*, *Mrpl23* and *Armcx5*. Among them, the differential expression trends of *Wnt7b*, *Gpr174*, *Ptbp3*, *Mrpl23*, *Armcx5* and *Raet1e* are consistent with the trends in GSE44770 dataset.

## Discussion

Microglia are tissue-resident macrophages in the central nervous system that have been shown to be activated in the ADs and close to the site of amyloid deposition 42. The effects of overactivated microglia on neurons and synapses may be negative. It is now generally accepted that M1 macrophages are thought to actively recruit to inflamed tissues and trigger pro-inflammatory innate immune responses 43. CD45^hi^ Ly6C^+^ CCR2^+^ monocytes could enter the CNS and modulate pathology in the context of disease 44. It has also been shown that senescent macrophages display a significant reduction in glycolysis and mitochondrial oxidative phosphorylation, which can lead to immune dysfunction 45. Moreover, curcumin can affect AD by enhancing macrophage-mediated clearance of Aβ 46. In the present study, by analyzing the GSE dataset of the GEO database, we determined the putative composition of 22 immune cells in the prefrontal cortex of 310 AD samples and 157 healthy samples. We constructed the co-expression network in that identified 14 different modules and found that the black module was highly associated with M1 macrophages and AD.

Based on LASSO and RF, we identified 10 hub genes associated with M1 macrophages and AD, including *ARMCX5*, *EDN3*, *GPR174*, *MRPL23*, *RAET1E*, *ROD1*, *TRAF1*, *WNT7B*, *OR4K2* and *ZNF543*. In an independent GSE44770 dataset, *GPR174*, *TRAF1*, *ROD1*,* RAET1E*,* OR4K2*,* MRPL23*,* ARMCX5* and *EDN3* were significantly different between the AD and healthy controls. We validated the results of bioinformatics analysis in 5XFAD transgenic mice, the relative mRNA levels of *Wnt7b*, *Gpr174*, *Ptbp3*, *Mrpl23*, *Armcx5*, *Traf1* and *Raet1e* were significantly different in AD and control groups. And the differential expression trends of *Wnt7b*, *Gpr174*, *Ptbp3*, *Mrpl23*, *Armcx5* and *Raet1e* are consistent with bioinformatics analysis. At the same time, these results also implied that the 5XFAD model has similarities but is not entirely consistent with human disease. *WNT7B,* a ligand of the Wnt signaling pathway, has been studied to demonstrate that dysregulation of the Wnt signaling pathway may be associated with synaptic failure and impaired cognitive function in neurodegenerative diseases 47. Wnt-7b increases presynaptic protein aggregation and synaptic vesicle recycling 48. It is suggested that alterations in common Wnt signaling pathways associated with early AD pathology and cognitive decline 49. *TRAF1*, TNF receptor associated factor 1, plays a key role in the immune system. It is regarded as a key signal transducers of many receptor families, such as innate immune receptors and adaptive immune receptors 50. SPRC (S-Propargyl-cysteine) has been shown to attenuate spatial learning and memory deficits via the TNF signaling and NF-κB signaling pathways in a rat model induced by lipopolysaccharide 51, but further studies are needed to elucidate the relationship between TRAF1 and AD. *ROD1*, the gene encodes an RNA-binding protein that was initially thought to act as a differentiation inhibitor 52, and later found to be a member of the heterogeneous nuclear ribonucleoprotein family. It also found to be involved in selective splicing of mRNA precursors 53. Recently, pyrophosphate sequencing analysis has shown that the methylation level of ROD1 is closely associated with aging in centenarians 54. *RAET1E*, raet1e (encoding Raet1) is a novel atherosclerotic gene 55. Both atherosclerosis and AD are thought to be associated with inflammation 56. But the relationship between *RAET1E* and AD has not been much studied, which may also be a potential target for AD research. *MRPL23*, mitochondria ribosomal protein L23, mitochondrial ribosomal protein (MRP) is an important component of the structural and functional integrity of the mitochondrial complex and has a major impact on the translational function of mitochondria 57. While the accumulation of damaged mitochondria is one of the causes of neurodegeneration in AD, and impaired mitochondrial autophagy has also been observed in the hippocampus of AD patients 58. But the direct relationship between *MPRL23* and AD remains to be determined by further studies. *GPR174* (G protein-coupled receptor 174) could induce rapid degranulation of mast cells 59, 60, limit proliferation of regulatory T cells 61 and enhance phagocytosis of apoptotic neutrophils by macrophages 62, 63. *ZNF543* (zinc finger protein 543), this gene has a wide range of physiological functions in a variety of cellular processes, which including apoptosis, cell proliferation and differentiation 64. *OR4K2* (olfactory receptor family 4 subfamily K member 2), olfactory receptor protein is a member of the large family of G protein-coupled receptors (GPCR) produced by a single coding exon gene. Olfactory receptors are responsible for recognition and G protein-mediated odor signaling. *EDN3*, Endothelin 3 is a powerful vasoconstrictor peptide in the adult enteric nervous system. EDN3 controls differentiation of enteric nervous system progenitors under the regulation of sox10 and ZEB2 65. *ARMCX5*, located on chromosome Xq22.1, a region associated with epilepsy 66, but there was no study in AD.

Of course, our study has some limitations. Firstly, the data we used was from a public database with a limited sample size, so it may not be a good representation of the true pathological state. Then, we did not validate the key genes *in vivo* and *in vitro* experiments. In addition, although we found that M1 macrophage is associated with AD, but the origin of M1 macrophage and relationship with AD need further studies to determine.

In conclusion, based on the expression matrix of AD and control, we initially explored the immune infiltration in the prefrontal cortex of AD patients and identified M1 macrophages and black module were associated with the occurrence of AD. We performed CIBERSORT and WGCNA to analyze the relationship between AD and immune cells for the first time. We identified 10 key genes associated with AD, which might be used as new targets for immunotherapy in AD patients.

## Figures and Tables

**Table 1:** Lists of primer sequences used for quantitative real-time PCR

Genes	Sequences
β-Actin	Forward: CTAAGGCCAACCGTGAAAAG
	Reverse: ACCAGAGGCATACAGGGACA
Ptbp3	Forward: CTCGCTTAGACCTTCCTACTGG
	Reverse: CTGCTTGAGGAAATGCGATGGC
Mrpl23	Forward: GTTTGCGGGACACCGAAAG
	Reverse: CCACCCAGTTGGTAAAGGGG
Armcx5	Forward: AAGAGCGCAGGTCCAACTTC
	Reverse: AGTCATTCAGCCCCTTTCCA
Wnt7b	Forward: ATGAGGACATGGGACCCTCA
	Reverse: AGCCCTGGCAGTTTCTTACC
Edn3	Forward: GCTGCACGTGCTTCACTTAC
	Reverse: GCTGGGAGCTTTCTGGAACT
Raet1e	Forward: ATCCTACCTCAGCAGACCTTC
	Reverse: TGGTGTTAGACACCTTGTCCC
Gpr174	Forward: TCTCCAAGGTAAGTGGTGCC
	Reverse: TGGCTGCTGGAATGATCCAC
Traf1	Forward: GCCCTGGACTGAGTTCCTATG
	Reverse: GAGGGGGACCCTGGGTATT

**Table 2:** The estimated coefficients for logistic least absolute shrinkage and selection operator (LASSO) regression between genes screened by the black module and AD

Variables	Coefficients
AARS	0
AASS	0
ABCB10	0
ABCC12	0
ABHD1	0
ABHD10	0
ACBD7	0
ADAM22	0
ADAM9	0
ADAMDEC1	0
DCY7	0
AMOTL1	0
AMPD1	0
AOC2	0
APAF1	0
APIP	0
ARIH2	0
ARMC7	0
ARMCX5	0.111768
C11orf40	0.13664
C1orf170	0
C21orf56	0
C6orf27	0.031063
C7orf21	0
C9orf21	0
C9orf52	0
C9orf75	0
CARD14	0
CASP2	0
CCDC15	0
CCDC50	0
CCDC8	-0.08756
CCPG1	0
CCRL1	0
CD99	0
CHRNA10	0
CLEC2D	0
COPS4	-0.18232
CR1L	0
CTAGE6	0
CTBS	0
CXCL9	0
CXorf27	0
CXorf36	0
DAAM2	0
DCT	0
DDIT4	0
DLEU1	0
DMWD	0
DNAH1	0
DNAH17	0
EDN3	0.069264
EGR3	0
ELK1	0
ELOVL2	0.014796
FAM20B	0
GPC6	0
GPR174	0.066463
GSTK1	-0.0569
GYG2	0
HCFC2	0
HCRTR2	0
HIST3H2BB	0
IRAK4	0
ITIH1	0
KCNH5	0.01496
KCNJ5	0
KLHDC8A	0
KLHDC2	0
KLHL17	0
KRT23	0
LASS3	0
LENG3	0
LIN7C	0
LMAN2	0
LMNB1	0
LRP1	0
LRP4	0
LRPPRC	0
MAPKAPK5	0
MBTPS2	0
MED28	0
MGLL	0
MGP	0
MGRN1	0
MRPL23	0.302214
MRPL30	0
MTHFD2	0
NALP12	0.100183
NDUFA3	0
NDUFB2	0
NLN	0
NMBR	0
NOS1AP	0.004739
NT5E	0
NTSR1	0
NUCB2	0
OR10AG1	0
OR13C3	0
OR2G2	0
OR4K15	0
OR4K2	-0.02666
PCDHGA8	0
PDRG1	-0.1436
PDXK	0
PF4	0
PKNOX2	0
PMS2L11	0
POLR2G	0
POMT2	0
POU6F2	0
PPIL2	0
PPP1R13B	0
PPP4R1L	0
PRR10	0
PSMA5	0
PTPLA	0
PXDN	0
PZP	0
RAB12	0
RAB17	0
RABAC1	0
RACGAP1P	
RAET1E	0.238109
RAMP1	0
RAPGEF1	0
RER1	0
RET	0
RFC2	0
RGS21	0
RHBDD1	0.047236
RIC8A	0
RNF170	0
ROD1	0.19671
ROS1	0
RSAD1	0
RTP2	0
STAT2	0
SCP2	0
SEC61B	0
SEH1L	0
SENP8	0
SERPINA13	
SETD7	0
SFTPA1	0
SGTB	0
SH3BGRL3	
0SHF	-0.16311
SLC17A5	
SLC23A2	
SLC25A22	0
SLC30A4	0
SLC35B4	0
SLC39A3	0
SLC3A1	0
SLC4A11	0
SLC6A14	0
SLCO1A2	0
SLCO2A1	0
SLITRK4	0
SMAD9	0
SMCHD1	0
SMCR2	0
SMOC2	0
SMPDL3B	0
SOD3	0
SORCS1	0
SOX5	0
SPIB	0
STAMBP	0
SUFU	0
SULT1C1	0
SYNPO	-0.16448
SYT10	0
SYT15	0
TAL1	0
TAS1R1	0.20351
TBC1D20	0
TBC1D19	0
TBCD	0
TESC	0.304705
THAP9	0
TICAM1	0
TLK1	0
TMC4	0
TMEM116	0
TMEM131	0
TMEM16K	0
TMEM47	0
TMEM80	0
TMEM93	0
TMPO	0
TNFAIP3	0
TNFRSF8	0
TNFSF18	0
TNK1	0
TNRC18	0
TP53I13	0
TRAF1	0.414105
TRAT1	0
TRIM8	0
TRO	0
TRPS1	0
TSPAN13	-0.15952
TSPAN18	0
TSSK6	0
TSTA3	0
TTC5	0
TUBGCP5	0
TUSC3	0
TXK	0
TXNIP	0
UBAP2	0
UBE2E4P	0
UBE2G1	-0.13186
UBE2W	0
UCP1	0
UNC119	0
UPF2	0
USP53	0
WDR48	0
WDR52	0
WIPI1	0
WNT7B	0.092085
WSB1	0
XPO4	0
YWHAQ	-0.01995
ZBTB34	0
ZCWPW2	0
ZMYND15	-0.04248
ZNF132	0
ZNF157	0
ZNF238	0
ZNF253	0
ZNF256	0
ZNF30	0
ZNF365	0
ZNF45	-0.08238
ZNF543	-0.09474
ZNF584	0
ZNF652	0
ZPBP2	0.097042

**Table 3:** The parameter of increase in node purity of top 30 gene based on RF analysis

	IncNodePurity
LRP1	11.36755
SYT10	5.41367
ROD1	5.029577
MRPL23	4.94144
TNFRSF8	4.006692
TRAF1	3.594842
WNT7B	3.361917
SPIB	3.326772
OR4K2	3.080312
GPR174	2.533245
SMCHD1	2.416868
RAET1E	2.407908
MED28	2.358533
AMPD1	2.312181
XPO4	1.717311
EDN3	1.659224
DMWD	1.470659
ZNF543	1.461082
ARMCX5	1.15924
SOX5	1.110552
HCFC2	1.031185
USP53	0.814927
RTP2	0.798482
KLHL17	0.682118
ITIH1	0.653343
WIPI1	0.588846
SLC35B4	0.584705
SLC6A14	0.582928
TNFAIP3	0.562013
TMPO	0.554761

**Table 4:** The confusion matrix index of logistic regression and k-fold cross-validation

Index	Logistic regression	k-fold cross-validation	
	test cohort	test cohort	training cohort
Precision	0.8936	1	0.8519
Recall	0.8400	0.8824	0.8241

## Abbreviations

AD: Alzheimer's disease
GEO: gene expression omnibus
WGCNA: weighted gene co-expression network analysis
LASSO: least absolute shrinkage and selection operator
RF: random forest
CNS: central nervous system
Tregs: T regulatory cells (TRegs)
GS: gene significance
MM: module importance
KEGG: kyoto encyclopedia of genes and genomes
ROC: receiver operating characteristic
TC-NER: Transcription-Coupled Nucleotide Excision Repair
AUC: area under the curve
MRP: mitochondrial ribosomal protein
GPCR: G protein-coupled receptors
